# Validity and reliability of the spiritual care competency scale for oncology nurses in Taiwan

**DOI:** 10.1186/s12904-022-00903-w

**Published:** 2022-02-04

**Authors:** Hui-Fen Fang, Henny Dwi Susanti, Lindelwa Portia Dlamini, Nae-Fang Miao, Min-Huey Chung

**Affiliations:** 1 Director of Administration Department, Taipei Cancer Center, Taipei Medical University, No. 250, Wu-Xing Street, 110 Taipei, Taiwan; 2 Deputy Director of Cancer Center, Taipei Medical UniversityTaipei Medical University Hospital, No. 252, Wu-Xing Street, 110 Taipei, Taiwan; 3 School of Nursing, College of Nursing, Taipei Medical University, No. 250, Wu-Xing Street, 110 Taipei, Taiwan; 4 Department of Nursing, Faculty of Health Science, University of Muhammadiyah Malang, Malang, East Java, Indonesia; 5 Post-Baccalaureate Program in Nursing, College of Nursing, Taipei Medical University, No. 250, Wu-Xing Street, 110 Taipei, Taiwan; 6 Department of Nursing, Taipei Medical University-Shuang Ho Hospital, Ministry of Health and Welfare, No. 291, Zhongzheng Rd., Zhonghe District, 23561 New Taipei City, Taiwan

**Keywords:** Nursing staff, Reproducibility of results, Spirituality

## Abstract

**Background:**

Nurses must have spiritual competence to provide holistic patient care. Therefore, the designed instrument to assess nurses’ competence could be a practical guide for health care professionals. This study aimed to evaluate the validity and reliability of the spiritual care competency scale (SCCS) for oncology nurses in Taiwan.

**Methods:**

This study used a convenience sample from a regional teaching hospital in Taiwan from November 2017 to February 2019, who were asked to complete the SCCS. We employed scale-content validity index (S-CVI). Exploratory Factor Analysis (EFA) was also used to evaluate the structural factor of SCCS. Confirmatory Factor Analysis (CFA) verified the construct validity of SCCS scale for oncology nurses in Taiwan. Test–retest reliability were also measured in this study at 2-week interval.

**Results:**

The average S-CVI of SCCS was 0.96. The EFA produced four factors of 27 items, such as professionalization, improving the quality of spiritual care, personal support, patient counseling and referral, attitude towards patient spirituality and communication, assessment, implementation providing and evaluation of spiritual care. Fitting the 27 items yielded an acceptable model fit; X^2^/df = 2.41, RMSEA = 0.08, GFI = 0.80, AGFI = 0.80, CFI = 0.92, IFI = 0.92, NFI = 0.90, RFI = 0.90, TLI = 0.91, SRMR = 0.06. Cronbach’s alpha values were between 0.93 and 0.95, and the total Cronbach’s alpha was 0.96. The intraclass correlation coefficient (ICC) scores were between 0.43 and 0.88.

**Conclusions:**

The result of this study demonstrated satisfactory validity and reliability for the SCCS in the nursing field in Taiwan. Implications for practice in this study serves as a reference for effectively evaluating nursing competency in spiritual care.

## Background

Nurses are intended to provide appropriate spiritual care to patients and their families [1]. In helping patients find meanings and purposes while in the hospital, spiritual care plays an important role [2]. Spiritual care is given to patients to meet their spiritual needs, in collaboration with other healthcare teams [1]. It begins with communication and dedication to caring for the patient relationships, such as paying respect to patients’ religious beliefs and culture [3], caring for patients with compassion, providing support, empathizing, and cooperating with other religious experts [2]. The spiritual care given to the patient is a central factor of holistic care [4]. However, it is not clear whether spiritual care was practiced properly in clinical settings and there is no proper instrument to access nursing competencies in spiritual care, especially oncology nurses.

Holistic nursing care provided to patients includes biopsychosocial and spiritual care. Lewinson et al. explained that providing spiritual needs to patients requires nursing competencies in spiritual care [5]. Competency is determined by the actions or performance of an individual and is evaluated as an indication of the level of capability to accomplish the task [6]. Nursing competencies in spiritual care produced six competencies include assessment and implementation of spiritual care, professionalization and improving the quality of spiritual care, personal support and patient counseling, referral to professionals, attitude towards the patient’s spirituality, and communication [7]. Previous research reported that even though patients expect spiritual care discussion, health care professionals express difficulty in implementing spiritual care for patients [8]. Other studies have also reported that patients’ spiritual needs are not met by their medical care team [9]. Time constraints, lack of knowledge and fear of assessing patients ‘spiritual needs made it difficult for health care professionals to meet and assess patients’ spiritual care [10, 11]. Therefore, health care professionals such as nurses still need to understand their spiritual care competency, as this is a key and a significant issue to be addressed, especially for oncology nurses.

An increasing number of patients in oncology wards and palliative care has called for the increased need for spiritually competent nurses. Nurses are often at the forefront of managing spiritual care while caring for dying patients in the oncology ward. Most nurses are aware of their obligation to provide spiritual care, but they often experience helplessness and confusion when caring for cancer patients. To ensure that spiritual care is appropriately practiced and a priority given to oncology patients, researchers first need a valid and reliable questionnaire to assess the competence of spiritual care. Spiritual instruments are designed to be guidelines for health professionals, such as nurses, to provide high-quality spirituality-related services to patients [12]. Studies have suggested that the success of assessment using psychometric instrument depends on the empathy and sensitivity of the nurses and is aimed to help health care professionals be more sensitive and aware of patients’ spiritual needs [7]. Several studies have performed tests of reliability and validity by using various scales to evaluate spiritual care. For instance, the Spirituality Scale for adults [13] and the Spiritual Orientation Scale for university students have been used in various faculties [14]. Another study was conducted in Turkey on spiritual care in nursing that evaluated nursing academics based on the Spirituality and Spiritual Care Rating Scale (SSCRS) [15]. Studies have assessed doctors, nurses, and midwives by using the Spiritual Support Perception Scale [16] and assessed nursing students by using the Spiritual Care-Giving Scale [17] and the Spiritual Care Competency Scale (SCCS) [18]. Countries that have developed the SCCS questionnaire were South Korea [19], China [20] ,and Brazil [21]. the SCCS questionnaire, because it can assess the competence of nurses in clinical service areas. The SCCS questionnaire can assess the competence of nurses in clinical service areas.

We chose the SCCS because it has good reliability and validity which can be used to assess spiritual care competence in different cultures and languages [20]. In past studies, the SCCS questionnaire was usually used to measure determinants related to spiritual care competence in Taiwan [10]. The qualitative study has used it to evaluate the perceptions and practices of spiritual care among doctors and nurses in hospitals in Taiwan [22]. For psychometric properties of the SCCS examined in East Asia such as China [20] and South Korea [19] in nursing practice, however, the target participants were general nurses and no exclusion criteria was applied [20]. Data for this study were from an online-based study which could affect participants’ answer due to the mobile-based interface [20]. In addition, in the Netherlands, nurses working in mental healthcare and home care reported higher spiritual care competency scores compared to hospital care nurses [23]. The perspectives and views on spiritual care of different ethnicities may differ [23]. However, there is currently no tool validated to access nursing competencies in spiritual care in Taiwan, especially for oncology nurses. In this study, we developed validity and reliability tests of spiritual care competency to assess nurses’ competencies in providing spiritual care, especially for patients with terminal illnesses, such as cancer, because these patients require physical, social, psychological, and spiritual support. This study aimed to identify the psychometric properties of the Taiwanese version of the SCCS in nursing practice.

## Methods

### Study design

This study used a cross-sectional design to verify the validity and reliability of the spiritual care competency scale.

### Participants

Respondents were recruited from a regional teaching hospital population in Taiwan through convenience sampling. The participants in this study were aged between 20 and 65 years old, with a nursing license or used to be nursing professionals. They’d been working as a registered nurse for at least 2 years and currently in an oncology ward, or worked with cancer patients in a palliative ward, medical-surgical ward, and gynecology ward. We excluded who were aged > 65 years and those working in the maternity ward or pediatric ward. The factor analysis requires at least five participants per item [24]. The SCCS questionnaire contains 27 items. By multiplying 27 items 5 times [24], we concluded that the minimum number of respondents needed in this study was 135. Moreover, for instrument evaluating, some experts recommended a sample of five to ten participants every item in the scale [25]. An adequate sample size was between 135 and 270 in this study. We invited and recruited 237 eligible participants, 22 of which had missing data, which yielded a total of 215 participants who completed the questionnaire and were included in this analysis. Moreover, we recruited additional 14 participants to examine test–retest reliability. Each participant completed the same questionnaire at a two-week interval. Test-retest reliability can be computed with at least 10 participants [26].

 This research was approved by the Taipei Medical University Joint Institutional Review Board (TMU-JIRB No. N201710014). Written informed consent in this study was obtained from participants. The research team used shift times to explain the research purpose and testing. The nursing staff who satisfied the requirements to receive the questionnaire were interviewed at an agreed time with respect for their rights and interests, or a questionnaire was anonymously self-completed and placed in the “question box of the questionnaire.” The research team then retrieved the data for analysis.

### Translation process

There are some differences between traditional Chinese version and simplified Chinese version. For instance, traditional characters are more complicated and have more strokes than simplified Chinese version. The translation of traditional Chinese version in this study comprised the following steps [27]: (1) After the permission from the author was obtained, the original version of the instrument was independently translated into traditional Chinese version by two translators who were fluent in Chinese and English. (2) Results of the two translated versions were compared, and a new translated version was prepared by synthesizing the phrases and terminology used by the two translators. (3) The final result of the translation was synthesized and retranslated back to English by two independent translators who had the same characteristics and qualifications as the first two translators. (4) The two back-translated questionnaires of SCCS were compared with the original questionnaire by four translators. (5) The questionnaire was distributed to 14 respondents to complete and evaluate the clarity, items, and questionnaire instructions. (6) Experts were invited to assess and provide feedback on the questionnaire. With at least 10 years of experience in their respective fields, three specialists and researchers with expertise in domestic spiritual care, hospice care, psychology and nursing contributed to the validity verification and evaluated the appropriateness and clarity of the four-point scoring method.

### Instrument of spiritual care competency scale (SCCS)

In the Netherlands, Van Leeuwen developed the SCCS [7]. The SCCS instrument consists of six subdomains, namely “assessment and implementation of spiritual care, professionalization and improvement of the quality of spiritual care, personal support and patient counseling, referral to professionals, attitude toward patients’ spirituality, and communication” [7]. Five-point Likert scales were used in the items where minimum and maximum scores were 27 and 135 respectively. Higher scores indicated higher nursing competencies in spiritual care. The Cronbach’s alpha of this study conducted in the Netherlands that used the original version in subdomain 1, 2, 3, 4, 5, and 6 were 0.82, 0.82, 0.81, 0.79, 0.56, and 0.71, respectively [7]. The original scale modified some terms “department” was revised into “ward”, “intervention” was changed into “peer discussion”, however, the original study did not measure the scale-content validity index [7].

### Statistical Analysis

#### Content Validity

These seven experts measured the value of the Content validity index (CVI) The content validity was categorized as relevant if the score of the average scale-content validity index (S-CVI) was ≥ 0.8 [28].

### Factor structure of the SCCS

In this study, a kurtosis and skewness value of less than 3 were calculated to indicate the normality of data. Exploratory factor analysis (EFA) was conducted to determine the main components in the correlation matrix of each item. When the correlation is higher, Promax allows factors to be correlated, otherwise, varimax assumed no intercorrelations between components. [29]. The Kaiser–Meyer–Olkin (KMO) test and Bartlett’s sphericity test were used to measure the adequacy of sampling and the accuracy of data for factor analysis [29]. A KMO greater than 0.8 was considered adequate enough to support the use of factor analysis [29]. The Bartlett’s test value of sphericity should be significant (*P* < 0.001) to indicate acceptability [30]. “A factor loading > 0.50 on the hypothesized component (factor) and < 0.30 on the other component were both set as evidence. Items with dual factor loadings > 0.40 were eliminated from the factor analysis” [31]. The number of factors was determined using variance explained criteria that accounted for 75–90% of the variance in the measured variables [32].

### Confirmatory factor analysis

We analyzed using Confirmatory factor analysis (CFA) for 27 questions and checked the result of Squared multiple correlation (SMC). Absolute fit indices were used to assess the fit of the model to the sample data [33] and indicate which model was used and which had the optimal fit [34]. The chi-square (χ^2^), goodness-of-fit index (GFI), root mean square error of approximation (RMSEA), and adjusted goodness-of-fit index (AGFI) were included in the absolute fit indices. Incremental fit indices (IFI) included “the normed fit index (NFI) and comparative fit index (CFI)”. GFI score and AGFI from 0.80 to 0.90 imply an acceptable model [35, 36].Value of IFI, Tucker Lewis Index (TLI), and CFI > 0.90 indicates acceptable model fit [37], for RMSEA < 0.10 [38]. A small root mean square residual (RMR) value indicates a good model [39].

### Convergent Validity and Discriminant Validity

The average variance extracted (AVE) value more than 0.50 is recommended [40], composite reliability (CR) score had to be more than 0.70. Furthermore, The result of all factor loadings had to be more than 0.70 to show that convergent validity was acceptable [41]. The square root of the AVE of each latent variable is greater than the correlation coefficients between that latent variable and other latent variables in the measurement model, then the model satisfies the discriminant validity criterion [42]. Second-order factors may be necessary to eliminate discriminant validity problems if the model fit of CFA does not guarantee discriminant validity [43].

### Reliability

This study used the intraclass correlation coefficient (ICC) to evaluate test–retest reliability. The ICC scores were between 0.43 and 0.88. ICC > 0.75 indicating excellent reliability and 0.4 > ICC < 0.75 showed acceptable reliability [44, 45]. The internal consistency of each subdomain of the SCCS was analyzed using Cronbach’s alpha; Cronbach’s alpha values of more than 0.70 indicate adequate internal consistency and sufficient reliability [46].

## Results

### Sample Characteristics

The mean age of participants in this study was 27.61 years with a standard deviation of 6.03. A majority of participants in this study were women (95.8%). Nearly all participants worked full time (97.7%). Most participants graduated from university (61.4%). Participants were Buddhist (25.1%), Taoist (28.8%), Christian (9.3%), Muslim (0.5%), and (36.3%) had other or no spiritual or religious affiliations.

### Content Validity Index

The item-content validity index (I-CVI) scores of all the items on SCCS questionnaire ranged between 0.67 and 1.00, with only 3 items with CVI (I-CVI) score of less than 0.80. Three items (number 2, 25, and 27) had value of 0.67 and made translational revisions about terms such as “in consultation with, personal limitation, and in my dealings with”; the others 24 items had a score of I-CVI equal to 1.00. On average, CVI (S-CVI) was 0.96. Even though, some of the I-CVI score were less than 0.8 (three items had a CVI of 0.67), there are still 2 out of 3 experts who gave high scores. We revised the content of the questionnaire based on the expert’s feedback and the principal investigation who is also expert in oncology nursing field, gave the final approval.

### Factor structure of the SCCS

**Fig. 1:**
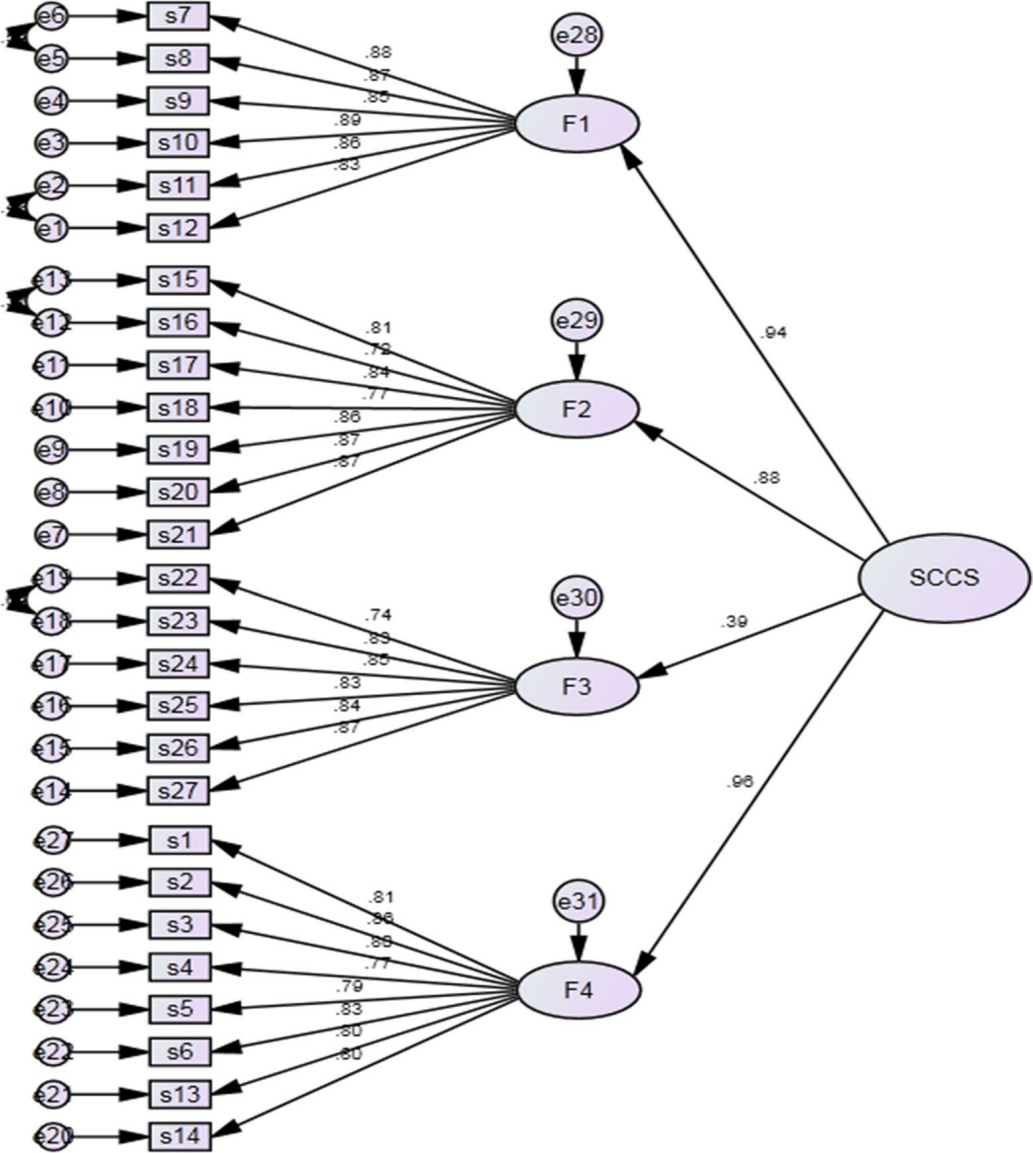
Scree plot

Our study used principal components for extraction method and promax for rotation method in the SCCS evaluated through EFA. Four factors explained 75.40% of the total variance. Twenty-seven items of SCCS remained after evaluating the number of factors in the SCCS instrument. The KMO test result for this study was 0.94, and Bartlett’s sphericity test value was statistically significant (P < 0.001). The percentage of variance and eigenvalue of the SCCS were 53.24% (14.37) for factor 1, 14.06% (3.79) for factor 2, 4.86% (1.31) for factor 3, and 3.25% (0.88) for factor 4. Based on the Scree plot, four factors were retained (Fig. 1). The factor loading value of all items for the four factors ranged from 0.71 to 0.89. The EFA of SCCS reinforced a four-factor structure, reproducing factor 1 professionalization and improving the quality of spiritual care (items s7, s8, s9, s10, s11, s12), factor 2 personal support, patient counseling and referral (items s15, s16, s17, s18, s19, s20, and s21), factor 3 attitude towards patient spirituality and communication (item s22, s23, s24, s25, s26, and s27), and factor 4 assessment, implementation, providing and evaluation of spiritual care (items s1, s2, s3, s4, s5, s6, s13, and s14).

### Convergent Validity and Discriminatory Validity

Table 1 presents the AVE scores, which ranged between 0.64 and 0.76. The AVE values indicated satisfactory convergent validity of the SCCS. The AVE values for factors 1, 2, 3 and 4 were 0.76, 0.67, 0.71, and 0.64, respectively. The CR was 0.95 for factor 1, 0.93 for factor 2, 0.94 for factor 3, and 0.93 for factor 4. The convergent validity value maintained by the CR results for every construct ranged between 0.93 and 0.95 and supported the convergent reliability of the SCCS instrument. The results of the square root of AVE were not higher than the values of the other correlated constructs; therefore, a second-order factor was used to eliminate discriminant validity problems in the results.

**Table 1:** Factor Loading, Convergent Reliability, and Convergent Validity (4-Factors)

Item no.	Dimension	Factor loading				AVE	CR
		F1	F2	F3	F4		
	**Factor 1 (Professionalization and improving the quality of spiritual care)**					0.76	0.95
s7	Within the nursing ward, I can contribute to quality assurance in the area of spiritual care	0.89					
s8	Within the nursing ward, I can contribute to professional development in the area of spiritual care	0.88					
s9	Within the nursing ward, I can identify problems relating to spiritual care in peer discussion sessions	0.79					
s10	I can coach other care workers in the area of spiritual care delivery to patients	0.88					
s11	I can make policy recommendations on aspects of spiritual care to the management of the nursing ward	0.89					
s12	I can implement a spiritual care improvement project in the nursing ward	0.88					
	**Factor 2 (Personal support, patient counseling and referral)**					0.67	0.93
s15	I can give a patient information about spiritual facilities within the care institution (including spiritual care, meditation centre, religious services)		0.79				
s16	I can help a patient continue his or her daily spiritual practices (including providing opportunities for rituals, prayer, meditation, reading the Bible/Koran, listening to music)		0.71				
s17	I can attend to a patient’s spirituality during the daily care (e.g. physical care)		0.83				
s18	I can refer members of a patient’s family to a spiritual advisor/pastor, etc. if they ask me and/or if they express spiritual needs		0.81				
s19	I can effectively assign care for a patient’s spiritual needs to another care provider/care worker/care discipline		0.84				
s20	At the request of a patient with spiritual needs, I can in a timely and effective manner refer him or her to another care worker (e.g. a chaplain/the patient’s own priest/imam)		0.87				
s21	I know when I should consult a spiritual advisor concerning a patient’s spiritual care		0.88				
	**Factor 3 (Attitude towards patient spirituality and communication)**					0.71	0.94
s22	I show unprejudiced respect for a patient’s spiritual/religious beliefs regardless of his or her spiritual/religious background			0.81			
s23	I am open to a patient’s spiritual/religious beliefs, even if they differ from my own			0.89			
s24	I do not try to impose my own spiritual/religious beliefs on a patient			0.86			
s25	I am aware of my personal limitations when dealing with a patient’s spiritual/religious beliefs			0.82			
s26	I can listen actively to a patient’s ‘life story’ in relation to his or her illness/handicap			0.82			
s27	I have an accepting attitude in my dealings with a patient (concerned, sympathetic, inspiring trust and confidence, empathetic, genuine, sensitive, sincere and personal)			0.84			
	**Factor 4 (Assessment, implementation, providing and evaluation of spiritual care)**					0.64	0.93
s1	I can report orally and/or in writing on a patient’s spiritual needs				0.83		
s2	I can tailor care to a patient’s spiritual needs/problems in consultation with the patient				0.84		
s3	I can tailor care to a patient’s spiritual needs/problems through multidisciplinary consultation				0.77		
s4	I can record the nursing component of a patient’s spiritual care in the nursing plan				0.76		
s5	I can report in writing on a patient’s spiritual functioning				0.81		
s6	I can report orally on a patient’s spiritual functioning				0.87		
s13	I can provide a patient with spiritual care				0.75		
s14	I can evaluate the spiritual care that I have provided in consultation with the patient and in the disciplinary/multidisciplinary team				0.74		

*Abbreviations: AVE* Average variance extracted; *CR* composite reliability

### Construct Validity

**Fig. 2:**
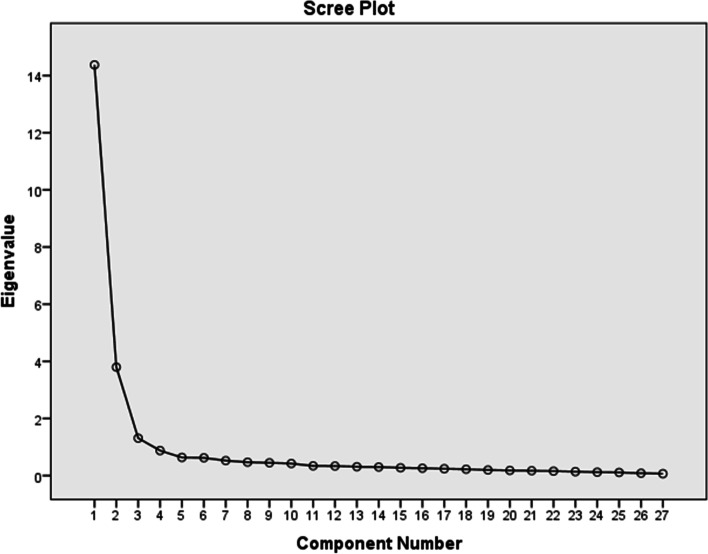
4-Factor Structure of the SCCS. Model fit index: χ2 /df = 2.41, RMSEA = 0.08, GFI = 0.80, AGFI = 0.80, CFI = 0.92, IFI = 0.92, NFI = 0.90, RFI = 0.90, TLI = 0.91, SRMR = 0.06

For the four-factors SCCS model, the model fit indexes were X^2^/df = 2.41, RMSEA = 0.08, GFI = 0.80, AGFI = 0.80, CFI = 0.92, IFI = 0.92, NFI = 0.90, RFI = 0.90, TLI = 0.91, Standardized root mean square residual (SRMR) = 0.06 (Fig. 2).

### Reliability

#### Internal Consistency

Table 2 presents the four subscales and the items related to each factor, item-total correlation and Cronbach’s alpha. The total Cronbach’s alpha for the SCCS was 0.96, and the values for each factor were: professionalization and improving the quality of spiritual care, personal support, patient counseling and referral, attitude towards patient spirituality and communication, and assessment, implementation, providing and evaluation of spiritual care were 0.95, 0.93, 0.93, and 0.94, respectively.

**Table 2:** Reliability analysis of Nursing Competencies for Spiritual Care

Item no.	mean	SD	Item-total correlation	Cronbach’s alpha	Test–Retest Reliability	
					ICC	95% CI
Factor 1				0.95	0.88	0.31-0.93
s7	3.29	0.76	0.79			
s8	3.31	0.78	0.80			
s9	3.41	0.74	0.79			
s10	3.26	0.80	0.77			
s11	3.07	0.83	0.75			
s12	3.01	0.85	0.74			
Factor 2				0.93	0.43	-0.77-0.82
s15	3.27	0.88	0.78			
s16	3.20	0.89	0.67			
s17	3.47	0.77	0.79			
s18	3.51	0.80	0.66			
s19	3.48	0.73	0.79			
s20	3.52	0.74	0.77			
s21	3.45	0.75	0.77			
Factor 3				0.93	0.54	-0.45-0.85
s22	3.96	0.75	0.48			
s23	4.03	0.73	0.43			
s24	4.12	0.79	0.39			
s25	3.92	0.74	0.48			
s26	3.95	0.74	0.57			
s27	4.09	0.69	0.45			
Factor 4				0.94	0.55	-0.42-0.85
s1	3.47	0.72	0.74			
s2	3.39	0.76	0.79			
s3	3.42	0.71	0.76			
s4	3.39	0.73	0.71			
s5	3.25	0.77	0.71			
s6	3.38	0.74	0.75			
s13	3.47	0.75	0.77			
s14	3.41	0.76	0.79			
Total				0.96		

Abbreviations: *ICC* intra-class correlation; *SD* Standard deviation

#### Test-retest reliability

Test–retest reliability was assessed using the ICC. It ranged from 0.43 to 0.88. Factor 1 was 0.88, factor 2 was 0.43, factor 3 was 0.54 and factor 4 was 0.55 (Table 2). The average of ICC from 4 variables was 0.6.

## Discussion

The present study is the first conducted in Taiwan on the development of instruments with validity and reliability to assess the SCCS in nurses who have experience in providing nursing care to patients with cancer. The Taiwanese version consists of 27 items with 4 factors. The methods used in this study showed that the Taiwanese version exhibited satisfactory construct validity, internal consistency and reliability.

A high factor value indicates that the item is related to that factor. The loading factor value of indicates poor was 0.32, fair is 0.45, good is 0.55, very good is 0.63, and 0.71 indicates excellent correlation [39, 47]. Based on EFA, our factor loadings for the four-factor scale ranged from 0.71 to 0.89. This was higher as compared to the Chinese version’s three-factor model (0.49 and 0.81) [20] and the Turkish version’s three-factor model, (0.436 to 0.895) [18]. In this study, the factor of all items met the criteria. Therefore, we maintained the 27 items similar to the original tool and in consistency with previous studies [7, 18, 48].

Reported factor structures have differed across countries. The present study identified 4 factors, contrary to the original six factors [7],the Chinese version’s three factors [20], and the Turkish version’s three factors [18]. These discrepancy in findings may possible be a result of differences in the perspective of spirituality that varies by country geographical location, populations, and culture [49–51].

In the present study, factor one which was called “Professionalization and improving the quality of spiritual care”, was made of six items, similar to the second factors of the original paper [7]. The name assigned was however inconsistent with the Chinese version which combined the first and second factors into factors one, and termed it “Assessment, implementation, professionalization and quality improvement of spiritual care” [20]. Our findings were also different from those found in Turkey, who called this factor “Assessment and implementation of spiritual care” [18]. Our analysis also showed that Factor two which was called “Personal support, patient counseling and referral”, had seven items, and was a combination of factor two and factor three of the original paper [7]. This was inconsistent with the previously reported Chinese version which combined factors three and four of the original paper and called this factor “Personal and team support” [20]. Our findings were also inconsistent with the Turkish version who combined three factors from the original paper, resulting in a total of 14 items in factor two, which they called “professionalization and patient counseling in spiritual care”[18]. Factor three in our study consists of six items and called “Attitude towards patient spiritually and communication”. It is a combination of factor five and six from the original paper [7]. This finding was consistent with the Chinese version [20] and the Turkish version [18] who called this factor “Attitude towards patient spirituality and communication. Factor four in our study combined factor one and four of the original paper [7], resulting in a total of eight items, which were called “Assessment, implementation, providing and evaluation of spiritual care”. Overall, the results of our study showed different number of factors from those of the original study.

This will enhance patient health by using the SCCS scale to provide services to patients, in particular spiritual care. Some of the challenges nurses face are lack of self-confidence, lack of awareness of spiritual care and perceptions of the patients’ inability to provide spiritual care. Nurses must have the skill and expertise as well as the spiritual care knowledge for patients in order to improve the service they give. Our study will help nurses identify their level of competence in spiritual care and as per need improve their skills in providing spiritual care to their patients. In order for nurses to have knowledge on spiritual care, the SCCS questionnaire can be developed and used as a nurse’s guide for assessing spiritual care competency, further we can provide of training protocols to train them. The Taiwanese version of the SCCS instrument has proven to be valid and reliable for nurses to evaluate their skills and competencies in meeting the spiritual needs of patients.

Our convergent validity of the SCCS was evaluated using AVE and CR. A CFA was performed to explore the construct validity of the SCCS. The values of the AVE and CR for the four factors demonstrated the high reliability and convergent validity of the SCCS. The CR results of our study indicated that all CR values from each subdomain were >0.70, which suggested high reliability and convergent validity for the SCCS instrument, and the correlation among the four factors of the SCCS was used to determine the discriminant validity of the SCCS instrument. The CFA result of our study indicated an acceptable model fit. Our findings were consistent with previous study in Chinese version that explained model fit [20].

For model fit of the CFA, some criteria of model fit for the CFA, should be required. RMSEA value less than 0.10 is acceptable [38]. Value of RMSEA in our study is 0.08, showed an acceptable model fit. Score of GFI and AGFI were 0.80 and 0.80, respectively, The GFI and AGFI indexes were not satisfactory, indicating that the model might not be parsimonious enough. This can be explained by model misspecifications, estimation method, and a smaller sample size [52]. However, the GFI and AGFI indexes reported in this study were still higher that those previously reported in the Chinese version; 0.78 and 0.74, respectively [20]. Similarly, our GFI index was also higher that the 0.76 reported in the Turkish version [18]. Moreover, for factor 6, more than three items are strongly recommended because for only two items, actually the model fit could not be assessed because the number of elements is tool small compared with the free parameters to be estimated [53]. However, Original paper did not evaluate using CFA for model fit, it only used item analyses explored EFA by PCA and varimax rotation, produced six-factors. Based on this, our study did not evaluate of 6-factors. With a CFI, IFI, TLI and NFI index of more than 0.9 [34, 38], the 4-factor SCCS model has a good fit of the data and was accepted for interpretation.

The factors in this study are similar to the previous factors. The scores on the item-total correlations in our study more than 0.30, indicating that the scale items were suitable [46]. The item-total correlation of each item revealed a strong correlation between the SCCS questionnaires and the total score, indicating the high reliability of the SCCS. The total Cronbach’s alphas in this study was 0.96. The Cronbach’s alphas for factor 1, 2, 3 and 4 were 0.95, 0.93, 0.93 and 0.94, respectively. The Cronbach’s alphas for the Taiwanese version showed to be higher than those reported in the original English version [7], but comparable to those reported in the Chinese version [20] and the Turkish version [18]. The ICC for four factors Taiwanese version from 0.4 to 0.75, that indicating the acceptable reliability of the SCCS [44, 45]. It means that SCCS is suitable for measurement survey of oncology nurses in Taiwan.

In view of these findings, our results cannot be generalized across countries, due to differences in ethnicity, culture, population, geography and nursing specialties. In addition, a self-report questionnaire-based was employed in this study, this may have caused information bias. Further research is needed to apply the SCCS questionnaire instrument especially to the clinical area and to carry out the validity and reliability of the questionnaire in different version.

### Implications for Clinical Practice

The SCCS demonstrates satisfactory results of validity and reliability. The SCCS instrument is effectively used to assess the competence of oncology nurses, which can be used as an indicator of the hospital to determine the quality of spiritual care in nurses. Further research may use this questionnaire to assess nursing competencies in spiritual care in the Chinese culture. Furthermore, we can assess the nurse’s competency score in spiritual care to provide the evidence that the scale obtained is consistent with nurses’ spiritual care level.

## Conclusions

The study showed that the reliability and validity of the SCCS possessed an acceptable model fit. This study serves as a reference for the SCCS in nursing practice. The SCCS instruments consist of four factors and 27 items. We recommend using this scale to test the expertise of nurses in clinical nursing, and to help nurses develop their skills in spiritual scale and in the field of education.

## Data Availability

The data used during this study are available from the corresponding authors on reasonable request.

## Abbreviations

AGFI: Adjusted goodness-of-fit index
AVE: Average variance extracted
CFA: Confirmatory factor analysis
CFI: Comparative fit index
CR: Composite reliability
CVI: Content validity index
EFA: Exploratory factor analysis
GFI: Goodness-of-fit index
ICC: Intraclass correlation coefficient
I-CVI: Item-content validity index
IFI: Incremental fit indices
KMO: Kaiser–Meyer–Olkin
NFI: Normed fit index
PCA: Principal component analysis
RMR: Root mean square residual
RMSEA: Root mean square error of approximation
S-CVI: Scale-content validity index
SCCS: Spiritual care competency scale
SSCRS: Spirituality and spiritual care rating scale
SMC: Squared multiple correlation
SRMR: Standardized root mean square residual
TLI: Tucker Lewis Index
